# A review on imaging techniques and quantitative measurements for dynamic imaging of cerebral aneurysm pulsations

**DOI:** 10.1038/s41598-021-81753-z

**Published:** 2021-01-26

**Authors:** L. B. Stam, R. Aquarius, G. A. de Jong, C. H. Slump, F. J. A. Meijer, H. D. Boogaarts

**Affiliations:** 1 Technical Medicine, University of Twente, Enschede, The Netherlands; 2 Department of Neurosurgery, Radboud University Medical Center, Radboud Institute for Health Sciences, Geert Grooteplein-zuid 30, Internal Post Number 633, Nijmegen, The Netherlands; 3 Department of Neurosurgery, Radboud UMC, Nijmegen, The Netherlands; 4 Technical Medical Center, University of Twente, Enschede, The Netherlands; 5 Department of Radiology and Nuclear Medicine, Radboud UMC, Nijmegen, The Netherlands

**Keywords:** Risk factors, Neurology

## Abstract

Measurement of intracranial aneurysm wall motion may refine the current rupture risk estimation. A golden standard for measuring aneurysm pulsation is lacking. The aim is to evaluate magnitudes of aneurysm pulsation as published in current literature. Embase and PubMed were searched for publications containing quantitative measures of cardiac-cycle related cerebral aneurysm pulsation (no date or language restrictions). Eleven studies were included, covering 197 unruptured and untreated cerebral aneurysms. Quantitative pulsation measurements were extracted from the studies. Characteristics of the study population and aneurysms were taken into account, as well as the imaging modality, scanning technique and data processing methods used. A meta-analysis was performed of studies with similar methodologies and individual IA measures and locations. The magnitude of the absolute volume pulsations varied between 14 ± 9 mm^3^ and 106 ± 123 mm^3^ and the mean relative volume change varied between 5 and 36%. The meta-analysis revealed a positive correlation between size and absolute volume change. The relative volume change in Basilar artery aneurysms seems smaller. No authors were contacted for original study data and articles only describing visual pulsations were excluded. The variation in methodologies impedes an accurate estimation of the magnitude of IA pulsations. Validation of aneurysm pulsation measurement is crucial prior to clinical studies evaluating IA pulsatility in relation to IA rupture risk. Prerequisite is a reliable and robust imaging method with high spatial and temporal resolution and standardization of the image analysis methods.

## Introduction

An intracranial aneurysm (IA) is a pathological, focal dilatation within the cerebral vasculature and rupture results in subarachnoid hemorrhage (SAH). SAH is fatal in 35–50% and almost half of the survivors suffer from long-term disability^1^. It is estimated that unruptured IAs are present in about 2–5% of the general population^2^.

The rupture risk of unruptured IAs can be removed or decreased through treatment. However, treatment is associated with a clinical complication risk, either transient or permanent morbidity or mortality, of 5.0% for endovascular treatment and 8.3% for neurosurgical treatment^3^. The estimated rupture risk of an IA is important in making the trade-off between the rupture risk of an unruptured IA and the risks associated with preventive treatment^1,4^.

The current rupture risk estimations are mainly based on static IA characteristics and patients factors. Unfortunately, patient history data shows frequent ruptures of IAs with an estimated low rupture risk^4^. Dynamical IA characteristics, like pulsatile wall motion, may expose the condition of the IA wall which could be an additional predictive factor for aneurysmal rupture^5^. A thinned, dark reddish wall is observed intra-operatively at the locations of pulsating blebs on pre-operative scans^6–8^. The presence of pulsating blebs was an independent risk factor for rupture in a cohort study of 168 intracranial aneurysms^9^. Furthermore, pulsating IAs are more likely to grow over time^10^. Growth is often seen as an intermediate for rupture, because the bleeding rate of growing IAs is approximately 10 times higher compared to stable IAs^11^. Increased wall motion could indicate locations with reduced stability and thus vulnerable sites for growth and/or rupture.

The magnitude of the IA wall pulsations are thought to be in the order of the resolution of the current imaging modalities and the reliability of pulsatility measurements is unknown^12^. The aim of this paper is to critically review current literature regarding quantitative measurement of IA pulsations, in order to reveal the magnitude of IA pulsations.

## Methods

### Search strategy and selection criteria

The protocol of this review was submitted to The International Prospective Register of Systematic Reviews (PROSPERO) on 30/3/2020 (ID 172044)^13^. PubMed and Embase were searched on 30/03/2020 for original study reports. Articles containing subsequent quantitative cerebral aneurysm motion measurements within one cardiac cycle were included. The search strategies were composed together with an experienced Liberian (Appendix A). No date or language restrictions were applied. The exclusion criteria were (1) No original study paper (i.e. conference abstracts, letters, case reports and reviews), (2) animal and ex-vivo studies, (3) Ruptured or treated cerebral aneurysms, and (4) No description of aneurysm measures of size, dimensions, volumes, morphology, amplitudes or wall motion/movement, over one or more cardiac cycles in the abstract.

The screening was performed by two reviewers (L.B.S. and R.A.) using the Rayyan online screening tool (https://rayyan.qcri.org, last accessed: 2020-04-05)^14^. Both reviewers were blinded to each other’s decisions. After each screening phase, conflicts were discussed and resolved.

### Data extraction

The data extraction was performed by one reviewer (L.S.) and verified by a second reviewer (R.A.). The following items were registered, if available, (1) bibliographical details (publication date, authors, journal), (2) imaging modality specifications *(imaging device, spatial resolution, temporal resolution, reconstruction method and the use of contrast)*, (3) post-processing methodology *(segmentation method, correlation with cardiac pulse wave pattern and additional filtering and correction methods)*, (4) study population and aneurysm characteristics *(patient’s age and gender , mean and maximal diameters of the aneurysms, location in cerebral vasculature, heart rate during measurement)*, and (5) main outcome *(measures indicating the relative and/or absolute volume change and deformations). *Both group summarizing values as well as individual values were extracted. In case the pulsatility measurements were displayed in graphs, the minimal and maximal values were determined and the relative volume change (*RVC*) was calculated by *RVC* $=\frac{Vmax-Vmin}{Vmin}*100\%$. If several methods were compared in one article, the best method according to the authors was extracted.

### Data analyses

The pulsatility outcomes were divided in absolute volume changes, relative volume changes and morphological changes. Furthermore, the individual location was assessed and the mean diameter of the IAs was calculated.

A meta-analysis was performed in case at least three studies satisfied the following conditions. A similar imaging modality was used, volume estimation was based on a 3D structure, individual mean or maximal aneurysm diameter were present and individual absolute or relative volume changes were present. The absolute volume changes were plotted against the aneurysm diameter. The Pearson correlation coefficient was determined for the absolute volume change. A p-value < 0.05 is considered statistically significant. Besides, the mean absolute and relative volume change was studied for the different IA locations.

## Results

**Figure 1:**
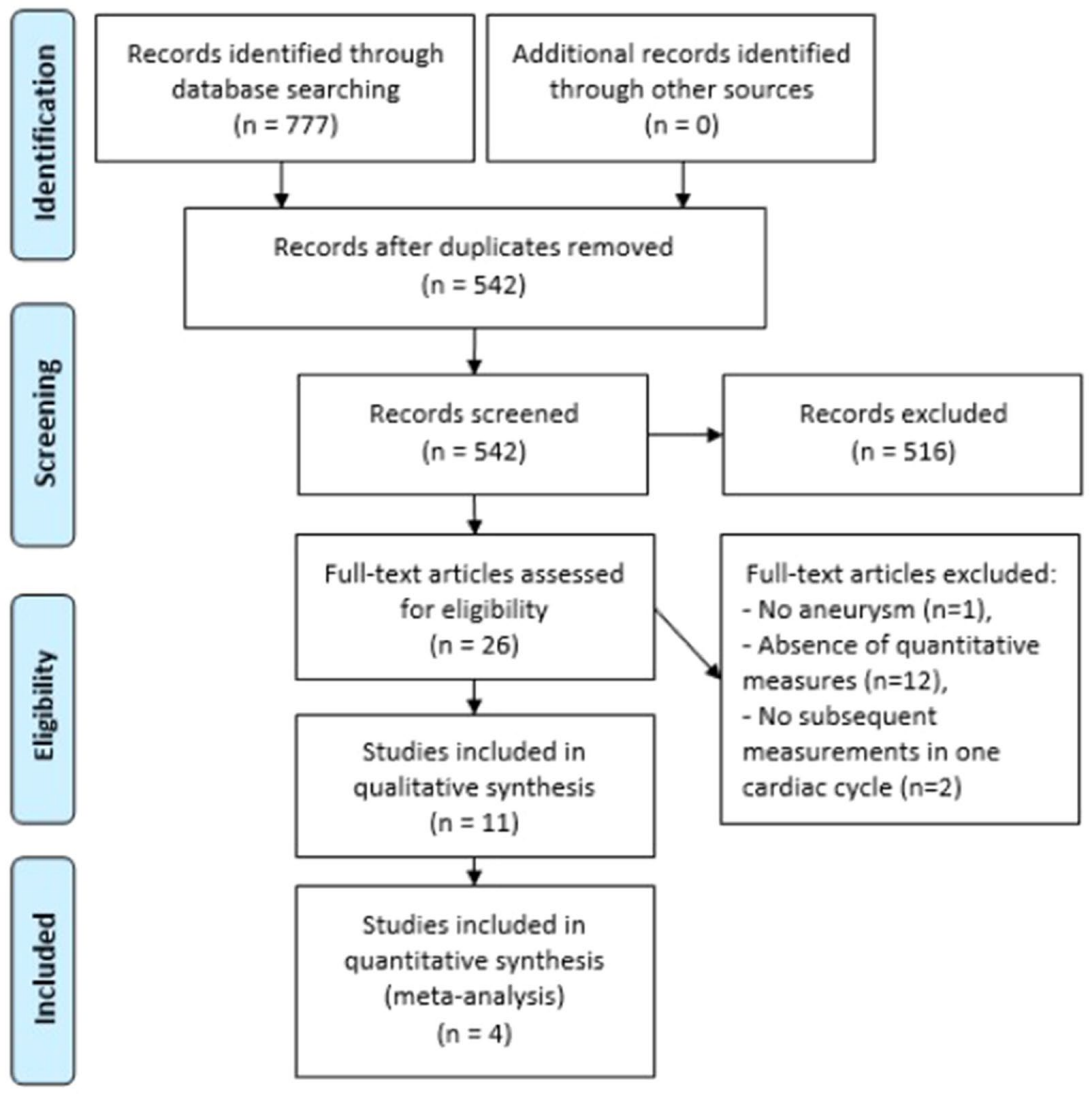
Prisma flow chart of the included studies^27^.

A Prisma flow chart is shown in Fig. 1. A total of 11 articles were included for the qualitative synthesis, of which four studies were included in the meta-analysis. Screening by two reviewers resulted in eleven conflicts. The summary of findings of the eleven included studies is shown in Table 1. The years of publication varied from 1993 to 2019. The used imaging modalities were Magnetic Resonance (MR) (3), Digital Subtraction Angiography (DSA) (1) and 4D Computed Tomography Angiography (4D CTA) (7). Spatial resolution is described as the 3 dimensional voxel dimensions and temporal resolution was expressed as the amount of phases per cardiac cycle. Studies published before 2012 performed volume measurements based on diameter changes, whereas studies from 2012 evaluated volume changes on the 3D structure. The main pulsatility measures were separated in IA volume changes and morphology changes.

**Table 1:** Summary of findings.

Study	Year pub	Imaging modality	3d based	Nr. of IAs	Mean diameter IAs (mm)	Volume pulsatility (mean ± std or median [95% CI])	Morphology changes
Meyer, Huston^18^	1993	MR (phase contrast)	No	10	10.3 ± 6.3	RVC = 17.6 ± 8.9% ΔV = 106.0 ± 123.5 mm^3^	
Oubel, Cebral^12^	2010	DSA	No	18	8.3 ± 5.7	μ* = 1.3%	Asymmetric deformations in width and depth
Karmonik, Diaz^17^	2010	MR (cine phase contrast)	No	7	10.0 ± 5.8		Local aneurysm distension and shrinkage
Kuroda, Kinoshita^22^	2012	4DCTA	Yes	22	6.9 ± 4.3	RVC = 5.4 ± 4.1% ΔV = 27.9 ± 60.5 mm^3^	
Firouzian, Manniesing^20^	2013	4DCTA	Yes	19	7.6 ± 3.7**	RVC = 8.0 ± 4.6%	Local aneurysm distension and shrinkage
Illies, Saring^15^	2014	4DCTA	Yes	10	8.1 ± 4.3**	RVC = 9.9 ± 8.8%	
Illies, Saering^5^	2016	4DCTA	Yes	14	9.1 ± 3.8	RVC = 8.3 ± 6.8%	
Kunitomi, Watanabe^21^	2016	4DCTA	Yes	8	10.9 ± 6.5	Coefficient of variation: 0.029 ± 0.014	
Gu, Zhang^28^	2018	4DCTA	Yes	Ir. 25, reg. 40	Ir. 5.3 ± 2.0, reg. 4.9 ± 1.6***	RVC irregular r = 36.8 ± 9.5%, regular = 29.0 ± 9.0%	
Kleinloog, Zwanenburg^16^	2018	MR (turbo field echo)	Yes	9	6.7 ± 2.7	RVC = 15 ± 11% ΔV = 14 ± 9 mm^3^	
Dissaux, Ognard^19^	2019	4DCTA	Yes	15	4.4 ± 2.8	RVC = 10.9% [4 17] ΔV = 16.4 [1.15 31.7] mm^3^	Variation in height, length and ostium change

Magnetic Resonance (MR), Digital Subtraction Angiography (DSA), 4D Computed Tomography Angiography (4D CTA), RVC = (Vmax − Vmin)/Vmin * 100%, ΔV = Vmax-Vmin, **Differential pulsation (μ) is the maximal change in IA depth or width, corrected for the artery diameter and the relative artery diameter change. **Max diameter instead of mean. ***Irregular shaped and regular shaped IAs were reported separately.

In three studies different reconstruction and data processing methods were compared, the result of the best method according to the author was extracted. The results of the AIDR3D method of Illies et al. were extracted instead of the filtered back projection results, the semi-automatic post-processing method of Illies et al. was selected above the manual method and in the study of Kleinloog et al., the results were selected of the phase in which the settings of turbo field echo sequence were improved and contrast was administered^5,15,16^.

### Imaging modality

Between 2012 and 2019, 7 studies performed quantitative pulsatility measurements with ECG-gated 4D Computed tomography angiography (4D CTA). The specifications of the 4D CTA scanners and reconstruction methods can be found in Appendix B. The spatial in-plane resolution varied between 0.3 and 0.4 mm and the slice thickness between 0.5 and 0.75 mm. The temporal resolution was 10 or 20 phases per heart cycle. The heart rates are displayed per study in Table C.1 (Appendix C).

In plane, isotropic spatial resolution of the three MR imaging studies varied from 0.6 mm (2018) to 1 mm (1993)^16–18^. Interpolation in phase direction was used to achieve a uniform spatial resolution in one study^17^. Both phase contrast and turbo field echo sequences were used to visualize blood flow and corresponding wall motion. The temporal resolution for MR techniques differed between 12 to 20 phases per heart rate. A gadolinium-based contrast agent was administered in one MR study^16^.

Only one study performed quantitative measurements on DSA. Spatial resolution varied between 0.071 and 0.283 mm. The temporal resolution varied between 2 to 60 frames per second^12^.

### Post-processing

The methods of image processing and aneurysm selection, are displayed in Table 2. Linear and B-spline sub-voxel interpolation were performed in some studies prior to the pulsatility measurements to achieve isotropic voxels and to measure magnitudes smaller than the CT resolution^17,19^. B-spline registration was applied in two studies to smoothen the image ^12,20^. Additional filtering was performed in the DSA study to decrease noise and grayscale inhomogeneities^17^.

**Table 2:** Overview of quality of scan, post-processing methods and main outcome.

Study	3D	Interpolation/filters	Segmentation	Corrections
Meyer, Huston^18^	No	–	–	
Oubel, Cebral^12^	No	B-spline registration	–	Varying contrast concentration
Karmonik, Diaz^17^	No	Interpolation, bandpass filter, edge preserving median filter	Single value	Vascular tree motion
Kuroda, Kinoshita^22^	Yes	–	110–890 HU → 1	–
Firouzian, Manniesing^20^	Yes	B-spline registration	time average + geodesic active contours	Deformation field of 8 mm
Illies, Saring^15^	Yes	–	160–890 HU → 1	–
Illies, Saering^5^	Yes	–	160–890 HU → 1	–
Kunitomi, Watanabe^21^	Yes	–	110–1400 HU → 1	–
Gu, Zhang^28^	Yes	–	–	–
Kleinloog, Zwanenburg^16^	Yes	–	–	–
Dissaux, Ognard^19^	Yes	B-spline interpolation	Semi-automatic	–

‘– ’ : no information was mentioned in the original article. Of Illies^15^ only the AIDR results are shown, of Illies^5^ only the semi-automatic, of Kunitomi the results from the APMC and of Firouzian only the CE-TFEi phase.

In order to determine the cardiac cycle-related volume change, a segmentation of the IA was created in studies published after 2010 to create 3D structures of which the volume was measured^5,15,21,22^. Cardiac cycle-related morphological changes were visualized using deformation fields in two studies^19,20^.

The influence of vascular tree motion was minimized in one study by a local coordinate system based on the aneurysm lumen location in each time frame^17^. Due to increasing contrast concentration, a low-frequency volume increase over a cardiac cycle was observed. This low frequency increase was removed with a high-pass filter^12^.

### Study population

In Table 3, the characteristics of the study population per study are shown. Except for Gu, all authors included the name of the adjacent vessel of the aneurysm. The locations of the IA varied widely between studies, as observed in Appendix C.

**Table 3:** Study population of the included studies.

Study	Nr. of IAs	Mean diameter (mm)	Max diameter (mm)
Meyer, Huston^18^	10	10.28 ± 6.34	–
Oubel, Cebral^12^	18	8.29 ± 5.73	–
Karmonik, Diaz^17^	7	10.01 ± 5.80	–
Kuroda, Kinoshita^22^	22	6.88 ± 4.33	–
Firouzian, Manniesing^20^	19	–	7.63 ± 3.67
Illies, Saring^15^	10	–	8.12 ± 4.27
Illies, Saering^5^	14	9.07 ± 3.75	–
Kunitomi, Watanabe^21^	8	10.89 ± 6.46	–
Gu, Zhang^28^—irregular	25	–	5.3 ± 2.0
Gu, Zhang^28^—regular	40	–	4.9 ± 1.6
Kleinloog, Zwanenburg^16^	9	6.69 ± 2.67	–
Dissaux, Ognard^19^	15	4.43 ± 2.78	–
Total	207		

‘—’: not mentioned.

### Pulsatility measurements

**Figure 2:**
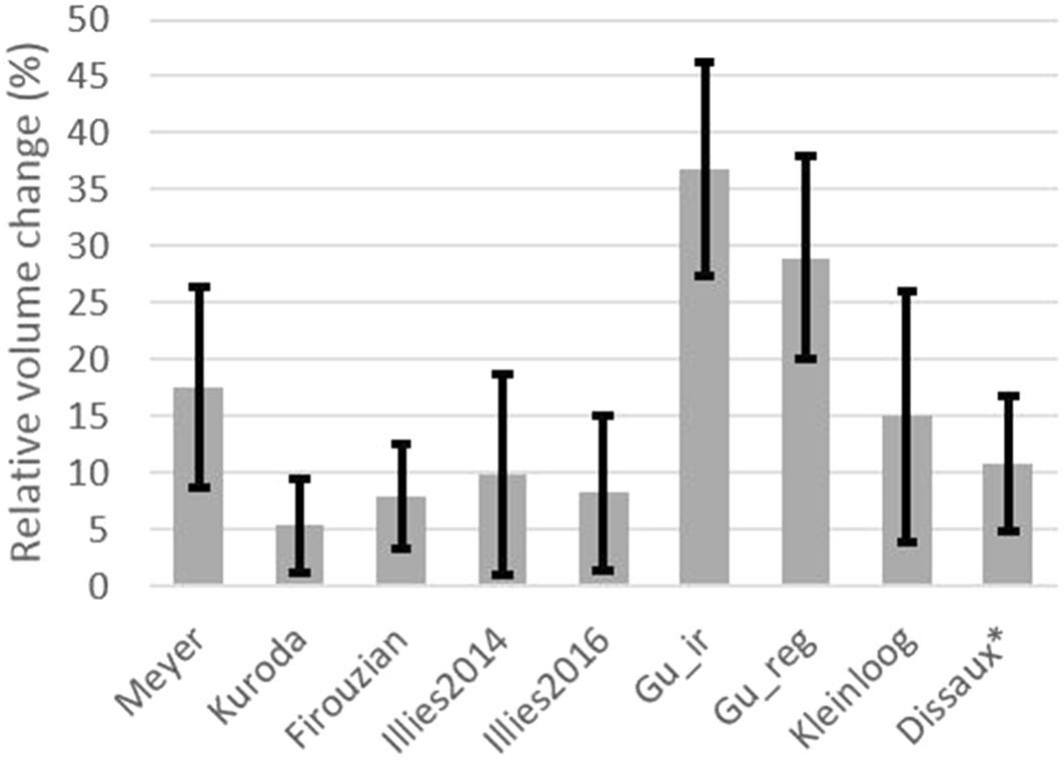
Mean relative volume change per study. Black error bars indicate the standard deviation. *The error bars represent the 95% CI-interval.

**Figure 3:**
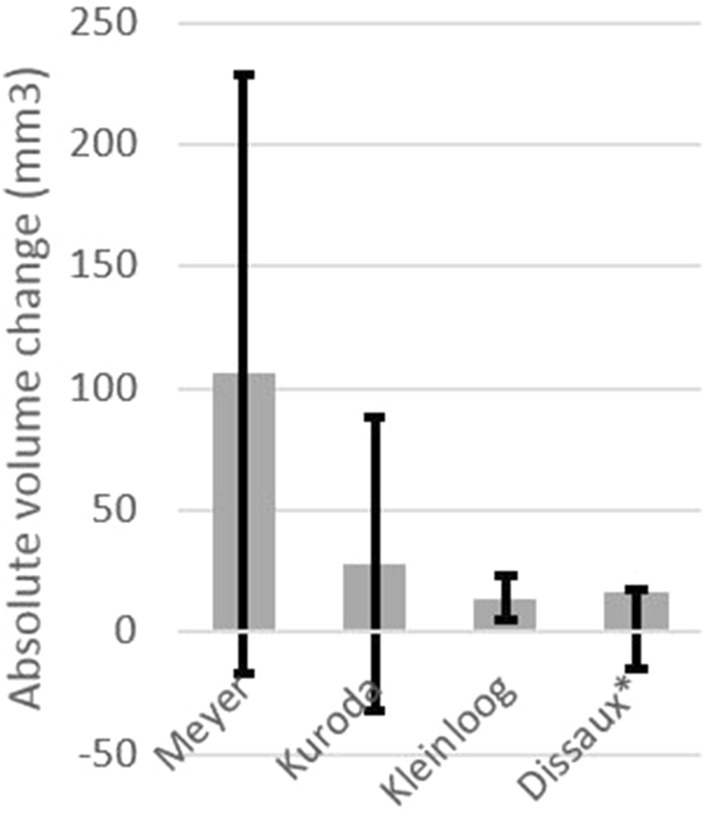
Mean absolute volume change per study. Black error bars indicate the standard deviation. *The error bars represent the 95% CI-interval.

The cardiac cycle-related volume change was measured in ten studies. The relative volume change was calculated in 8 studies and the mean RVC varied between 5.4 ± 4.1% and 36.8 ± 9.5%. In Fig. 2, the mean relative volume change and standard deviation per study were visualized. Absolute volume change (ΔV) was calculated in four studies and varied between 14 ± 9 mm^3^ and 106 ± 123 mm^3^. The mean and standard deviation per study are visualized in Fig. 3. Cardiac cycle-related morphological changes were described in four studies and non-uniform motion in different dimensions and locations of the dome was observed.

### Meta-analysis

Four studies using 4D CTA were included in the meta-analysis and the summary is shown in Table 4. Differences in resolution, segmentation methods and diameter definitions were observed.

**Table 4:** All studies included in meta-analysis.

First author	Resolution [mm]	Segmentation	Nr. Of IAs	Size	Maximal diameter (mm)
Firouzian, Manniesing^20^	0.4 × 0.4 × 0.75	Time aver. + geodesic active contours	19	Maximal diameter	7.63 ± 3.67
Illies, Saering^5^	0.39 × 0.39 × 0.5	160–890 HU → 1	14	Mean axial plane	9.07 ± 3.75
Kunitomi, Watanabe^21^	0.39 × 0.39 × 0.5	110–1400 HU → 1	8	Mean of 2 dimensions	10.89 ± 6.46
Dissaux, Ognard^19^	0.30 × 0.30 × 0.5	Semi-automatic	15	Mean height	4.43 ± 2.78

**Figure 4:**
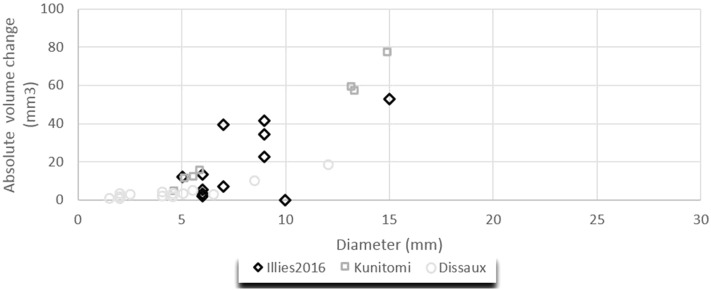
Absolute volume change of aneurysm with certain diameters.

In Fig. 4, the absolute volume change is visualized against the aneurysm diameter. The Pearson correlation coefficient revealed statistically significant positive correlations between the aneurysm diameter and absolute volume change in the studies of *Illies* 2016 (r = 0.81, P = 0.0003), *Kunitomi* (r = 0.88, P = 0.002) and *Dissaux* (r = 0.90, P < 0.0001).

**Figure 5:**
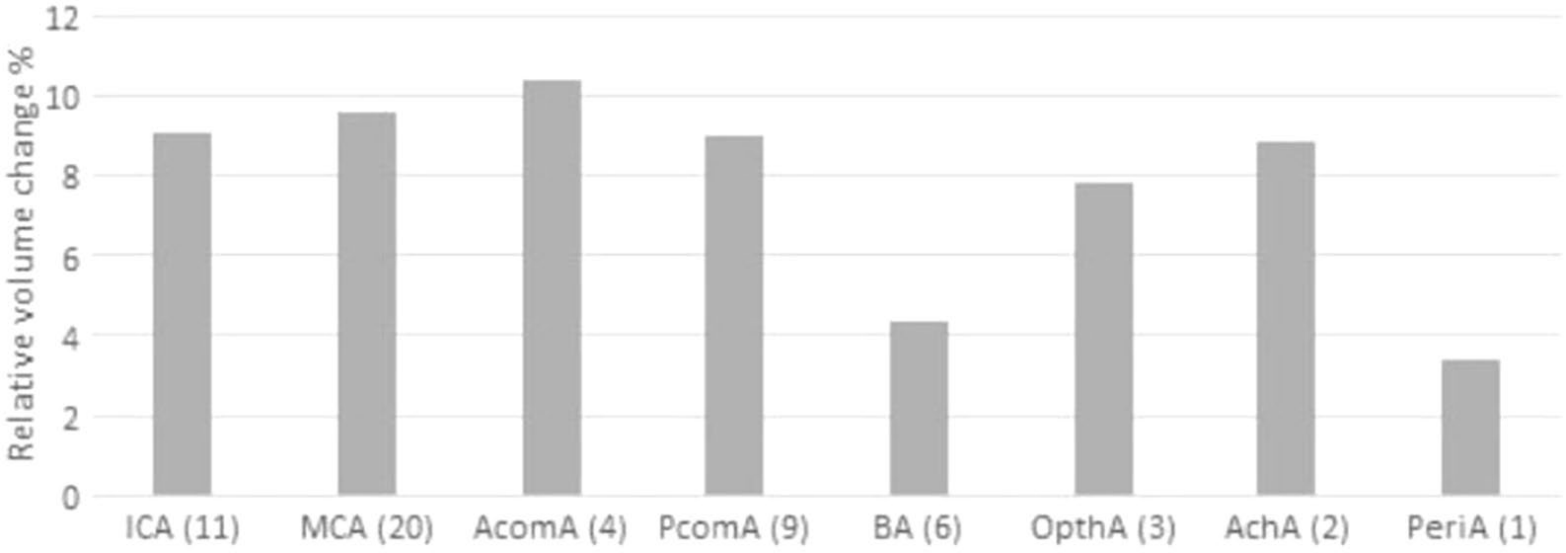
Mean relative voume change per aneurysm location. Error bars indicate the standard deviation. ICA = Internal Carotid Artery, MCA = Middel Cerebral Artery, AcomA = Anterior communicating Artery, PcomA = Posterior communicating Artery, BA = Basilar Artery, OpthA = Opthalamic Artery, AchA = Anterior choroid Artery, PeriA = Pericallosal Artery.

In Table 5, the mean size, absolute and relative volume change per location are listed for the four studies. In Fig. 5, the mean relative volume change is shown for each location.

**Table 5:** Mean size, absolute and relative volume change per aneurysm location.

Location (nr. of IAs)	ICA (11)	MCA (20)	AcomA (4)	PcomA (9)	BA (6)	OpthA (3)	AchA (2)	PeriA (1)
Mean diameter (mean ± std)	7 ± 4	9 ± 5	5 ± 2	6 ± 2	9 ± 5	8 ± 3	6 ± 1	4
Absolute volume change [mm^3^] (mean ± std)	20 ± 22	50 ± 106	8 ± 3	13 ± 22	18 ± 14	24 ± 13	6 ± 2	1
Relative volume change [%] (mean ± std)	9 ± 6	10 ± 7	10 ± 5	9 ± 6	4 ± 2	8 ± 5	9 ± 5	3

*ICA* internal carotid artery, *MCA* middel cerebral artery, *AcomA* anterior communicating artery, *PcomA* posterior communicating artery, *BA* basilar artery, *OpthA* opthalamic artery, *AchA* anterior choroid artery, *PeriA* pericallosal artery.

## Discussion

Current literature was reviewed on quantitative measurements of IA pulsations, in order to determine the magnitude of these pulsations. The cardiac-cycle related volume change was reported in 10 studies. The magnitude of the cardiac-cycle related volume pulsations varied between 14 ± 9 mm^3^ and 106 ± 123 mm^3^ and the mean relative volume change varied between 5 and 36%. The meta-analysis revealed a positive correlation between the aneurysm diameter and the absolute volume change. The relative volume change seemed smaller for Basilar artery IAs. Quantitative morphological changes during a cardiac cycle were described in four studies and non-uniform motion in different dimensions and locations of the dome was observed.

A wide variation in absolute and relative volume changes has been reported. The major difficulty for interpretation and comparison of the diverse study results is that a reliable golden standard is missing. Therefore, it can be debated whether true IA pulsation is measured, or that variations in aneurysm diameters should be attributed to image noise and measurement errors. Differences in imaging modalities, scanning techniques and post-processing methods hamper the interpretation and comparison of the study results.

First, the selection of imaging modality, scanning technique and image reconstruction is crucial in the minimization of these possible fictitious pulsations. A combination of high spatial and temporal resolution is required. 4D-CTA is advantageous over MR as MR is limited by a trade-off between spatial and temporal resolution. The variation in spatial and temporal resolution between studies may account for variations in measured pulsations. The four dimensionality of MR and 4D CTA enables the visualization of the complete dome over the cardiac cycle, whereas the DSA only capture one projection per time frame. There is a risk that local distension or shrinkage was missed by the DSA if the projection direction was not suited for the detection of local motion. Filtering and interpolation could decrease partial volume effect and image noise^12,17,19,20^.

Second, the method of image segmentation and measurements are probably of influence on the observed volume pulsations. Meyer et al. calculated the volume based on diameters in three orthogonal directions, missing information about regions outside these directions^18^. The segmentation of the IA dome enables volume calculation based on a 3D structure. However, all studies performed the segmentation (partly) manually, leading to high inter-reader variability^5,19^. Automation of aneurysm segmentation could reduce intra- and interrater variability. Firouzian et al., selected only the lumen center manually, decreasing the risk of inter-reader variability^20^. The lack of a uniform pulsatility measure has led to various main outcome measures, hindering comparison. Even the diameter measurements changed widely, the horizontal position could be displaced due to this variation in diameter measurements.

Third, IA characteristics may account for the high intra- and interstudy variability in the magnitude of pulsation. If the positive correlation between aneurysm size and absolute volume change is applicable for all aneurysms, studies containing larger IAs should show larger absolute volume pulsations. This could account for the difference in absolute volume change between Kuroda et al. and Dissaux et al.^19,22^. The locations in the cerebral circulation of the aneurysms may have caused variation in pulsatility^20^. Due to the small number of IAs per location subgroup, no statistical analysis could be applied. However, the relative pulsation seemed smaller for Basilar artery aneurysms. Furthermore, it can be speculated to what extent the underlying vessel wall pathology and comorbidity are of influence.

Finally, wall motion does not have to lead to volume changes, as shown by four different methods for analyzing the morphodynamical IA behavior^12,17,19,20^. Visualizations of morphological changes, such as deformation fields, enable comprehensive analysis per aneurysm of the locations with increased pulsatility^20^. Quantification of wall deformities is performed by Oubel et al., by calculating the maximal distension or shrinkage per point of the dome^17^. By calculation the extreme vales and the standard deviation of the dome, morphodynamical information was summarized. Evaluating the morphodynamical behavior is essential in the detection of locations with reduced stability.

There are several limitations in this review. No authors were contacted for original study data. Original study data enables measuring uniform sizes between various studies, which could have expanded and improved the meta-analysis. Furthermore, qualitative studies with visually detected pulsations are excluded however, these studies could uncover the presence of pulsating blebs^7^. Deformations over the cardiac cycle are likely to indicate locations with reduced stability. Qualitative studies showed the correlation between the location of a pulsating bleb and the intra-operative rupture location^6,10^.

Validation of the vessel wall pulsation measurements is crucial in order to evaluate the additional value for rupture risk estimations. An ex vivo phantom experiments should be performed to determine the accurateness and precision of pulsatility measurements. A potential validation method may be the comparison of a known volume increase with the observed dimension change. Other recommendations for future research are listed below. Firstly, the spatial as well as the temporal resolution of the imaging modality should be as high as possible. The new generation 4D CTA scanners are a promising imaging modality, due to the submillimeter spatial resolution and the convenient temporal resolution^23^. The Nyquist theorem, applied on a pulsatile motion similar to the cardiac pulse wave, lead to a required sampling frequency of minimal 8 Hz^12^. The effect of the heart rate, contrast administration and different reconstruction methods and the possibility for noise reduction should be considered by composing the scan protocol. Secondly, noise-reducing post-processing methods, like smoothing filtering, should be used. Automation of post-processing aspects could objectify the results and could reduce the time needed for pulsatility analysis. Quantitative and uniform measures of IA pulsatility are required, which should uncover locations with decreased stability. A combination of several measures is required to analyze the pulsatile wall motion. The standardization of measurements and studies based on larger (> 100 IAs) databases may answer the question which pulsatility outcomes bring along increased rupture risks. Uniform measures would enable comparison between subgroups, for example to study the effect of size and location on the IA pulsatility. Furthermore, computational flow dynamics models can clarify the relation between the pulsatility pattern and the cardiac pulse wave^5^. Overall the combined measures of the pulsatility pattern and similarities with the cardiac pulse wave, the combination with vessel wall MR, biomarkers and computational flow dynamics may provide a more reliable estimate of the stability of the aneurysm wall^24–26^.

## Conclusion

This review critically evaluated current literature on quantitative cardiac-cycle related pulsatility measures of IAs. The variation in outcome measures, methodology, study population and study quality impedes an accurate estimation of the magnitude of cardiac-cycle related volume variations and deformities. Validation of aneurysm pulsation measurement is crucial prior to clinical studies evaluating IA pulsatility in relation to IA rupture risk. Prerequisite is a reliable and robust imaging method with high spatial and temporal resolution and standardization of the image analysis methods.

## Supplementary Information

Supplementary Information 1.

Supplementary Information 2.

Supplementary Information 3.
