# Supplementary material for: A review on imaging techniques and quantitative measurements for dynamic imaging of cerebral aneurysm pulsations
Source: Sci Rep. 2021 Jan 26;11:2175. doi: 10.1038/s41598-021-81753-z (PMC7838168; doi:10.1038/s41598-021-81753-z)
Supplement: Supplementary file 1 — Supplementary Information 1. [file 41598_2021_81753_MOESM1_ESM.docx]

A Review on Imaging Techniques and Quantitative Measurements for Dynamic Imaging of Cerebral Aneurysm Pulsations

*November 3, 2020*

L. B. Stam^1*^, R. Aquarius^2^, G. de Jong^2^, C. H. Slump^3^, F. J. A. Meijer^4^, H. D. Boogaarts^2^

^1^ Technical Medicine, University of Twente, Enschede, The Netherlands. ^2^ Department of Neurosurgery, Radboud UMC, Nijmegen, The Netherlands. ^3^ Technical Medical Center, University of Twente, Enschede, The Netherlands. ^4^ Department of Radiology and nuclear medicine, Radboud UMC, Nijmegen, The Netherlands.

*Corresponding author. L. B. Stam

Department of Neurosurgery

Radboud University Medical Center

Geert Grooteplein-zuid 30

Internal post number 633

Nijmegen

The Netherlands

[Lotte.stam@radboudumc.nl](mailto:Lotte.stam@radboudumc.nl)

### Appendix A: search strategies

30^th^ of march, 2020

|  | **Pubmed** | **Embase** |
| --- | --- | --- |
| 1 | ("Intracranial Aneurysm"[Mesh] OR ("Aneurysm"[Mesh:NoExp] AND ("Brain"[Mesh] OR "Meninges"[Mesh] OR "Cerebral Arteries"[Mesh])) OR ((intracranial[tiab] OR brain[tiab] OR cerebral[tiab] OR berry[tiab] OR saccular[tiab]) AND aneurysm[tiab]) OR ((basilar[tiab] OR anterior communicating[tiab] OR posterior communicating[tiab] OR anterior cerebral[tiab] OR middle cerebral[tiab] OR posterior cerebral[tiab] OR internal carotid[tiab]) AND artery[tiab] AND aneurysm*[tiab])) | exp intracranial aneurysm/ OR ((intracranial.ti,ab,kw. OR brain.ti,ab,kw. OR cerebral.ti,ab,kw. OR berry.ti,ab,kw. OR saccular.ti,ab,kw.) AND aneurysm.ti,ab,kw.) OR ((basilar.ti,ab,kw. OR anterior communicating.ti,ab,kw. OR posterior communicating.ti,ab,kw. OR anterior cerebral.ti,ab,kw. OR middle cerebral.ti,ab,kw. OR posterior cerebral.ti,ab,kw. OR internal carotid.ti,ab,kw.) AND artery.ti,ab,kw. AND aneurysm*.ti,ab,kw.) |
| 2 | ("diagnostic imaging" [Subheading] OR "Diagnostic Imaging"[Mesh] OR Imaging[tiab] OR Radiograph*[tiab] OR Ultrasound[tiab] OR MRI[tiab] OR MRA[tiab] OR "Magnetic resonance"[tiab] OR Optical[tiab] OR Ct[tiab] OR CTa[tiab] OR computational tomograph* [tiab] OR Computed tomograph*[tiab] or angiograph*[tiab] OR Dsa[tiab] OR ecg-gated[tiab]) | exp "imaging and display"/ or Imaging.ti,ab,kw. OR Radiograph*.ti,ab,kw. OR Ultrasound.ti,ab,kw. OR MRI.ti,ab,kw. OR MRA.ti,ab,kw. OR "Magnetic resonance".ti,ab,kw. OR Optical.ti,ab,kw. OR Ct.ti,ab,kw. OR CTa.ti,ab,kw. OR computational tomograph*.ti,ab,kw. OR computed tomograph*.ti,ab,kw.OR angiograph*.ti,ab,kw. OR Dsa.ti,ab,kw. OR ecg gated.ti,ab,kw. |
| 3 | 1 AND 2 | 1 AND 2 |
| 4 | (Pulsatile flow[mesh] OR pulsa*[tiab] OR pulse wave*[tiab] OR motion[tiab] OR movement[tiab] OR Volume change[tiab] OR Cardiac cycle*[tiab] OR Volume variation[tiab] OR Cardiac phase*[tiab]) | Exp pulsatile flow/ or exp motion/ or exp movement/ or (pulsa*.ti,ab,kw. OR pulse wave*.ti,ab,kw. OR motion.ti,ab,kw. OR movement.ti,ab,kw. OR (Volume change or Cardiac cycle* or Volume variation or Cardiac phase*).ti,ab,kw.) or heart cycle/ |
| **5** | **3 AND 4** | **3 AND 4** |
| 6 | **"Case Reports" [Publication Type] OR case report[ti]** | Case report/ or case report.ti. |
| 7 | 5 NOT 6 | 5 not 6 |
| 8 |  | Limit 7 to conference abstracts |
| 9 |  | 7 not 8 |
