# Supplementary material for: A review on imaging techniques and quantitative measurements for dynamic imaging of cerebral aneurysm pulsations
Source: Sci Rep. 2021 Jan 26;11:2175. doi: 10.1038/s41598-021-81753-z (PMC7838168; doi:10.1038/s41598-021-81753-z)
Supplement: Supplementary file 2 — Supplementary Information 2. [file 41598_2021_81753_MOESM2_ESM.docx]

A Review on Imaging Techniques and Quantitative Measurements for Dynamic Imaging of Cerebral Aneurysm Pulsations

*November 3, 2020*

L. B. Stam^1*^, R. Aquarius^2^, G. de Jong^2^, C. H. Slump^3^, F. J. A. Meijer^4^, H. D. Boogaarts^2^

^1^ Technical Medicine, University of Twente, Enschede, The Netherlands. ^2^ Department of Neurosurgery, Radboud UMC, Nijmegen, The Netherlands. ^3^ Technical Medical Center, University of Twente, Enschede, The Netherlands. ^4^ Department of Radiology and nuclear medicine, Radboud UMC, Nijmegen, The Netherlands.

*Corresponding author. L. B. Stam

Department of Neurosurgery

Radboud University Medical Center

Geert Grooteplein-zuid 30

Internal post number 633

Nijmegen

The Netherlands

[Lotte.stam@radboudumc.nl](mailto:Lotte.stam@radboudumc.nl)

### Appendix B: 4D CTA specifications

**Table B.1.** Specifications of studies performed with 4D CTA. *Gu did only mention the voxel volume of 0.1 mm^3^, the values are derived in case of a isotropic voxel.

FBP: filtered back projection, hs rec.: halfscan reconstruction, icv kernel: kernel for intracranial vessels.

| 4DCTA | CT Device | Inplane resolution [mm] | Slice [mm] | Phase interval | tube voltage (kV) | tube Current | Rad. dose | Contrast [mgI/mL], inj.speed [ml/s] | Reconstruction/ kernels |
| --- | --- | --- | --- | --- | --- | --- | --- | --- | --- |
| **Kuroda et al.** | Aquilion ONE | - | 0.5 | 10% RR | 120 | 270 mA | - | Optiray 320, 5 | icv kernel |
| **Firouzian et al.** | Siemens Somatom definition | 0.4x0.4 | 0.75 | 5% RR | 120 | 160 mAs/rot | 0.3-0.8 mSv | Iodixanol 320, 4 | intermediate sharp (B31F) |
| **Illies et al. (2014)** | Aquillion ONE | 0.39x0.39 | 0.5 | 10% RR | 120 | 270 mA | - | Optiray 320, 5 | FBP hs rec. + icv kernel |
|  |  |  |  |  |  |  |  |  | AIDR3D |
| **Illies et al. (2016)** | Aquilion ONE | 0.39x0.39 | 0.5 | 10% RR | 120 | 270 mA | - | Optiray 320, 5 | FBP icv kernel +  AIDR3D |
| **Kunitomi et al.** | Aquilion ONE | 0.39x0.39 | 0.5 | 10% RR | 120 | 270 mA | - | Optiray 320, 5 | AIDR3D + hs rec. |
|  |  |  |  |  |  |  |  |  | AIDR3D + APMC |
| **Gu et al.** | Siemens Somatom definition | ~0.46* | ~0.46* | 5% RR | 120 | 110 mA | 0.3 ± 0.1 mSv | iohexol 370, 4.5 | Coronary artery CTA mode |
| **Dissaux et al.** | Aquilion ONE | 0.3x0.3 | 0.5 | 10% RR | 120 | 230 mA | 0.6 mSv | Iomeron 400, 4 | FC43 with AIDR3D |
