# Supplementary material for: A review on imaging techniques and quantitative measurements for dynamic imaging of cerebral aneurysm pulsations
Source: Sci Rep. 2021 Jan 26;11:2175. doi: 10.1038/s41598-021-81753-z (PMC7838168; doi:10.1038/s41598-021-81753-z)
Supplement: Supplementary file 3 — Supplementary Information 3. [file 41598_2021_81753_MOESM3_ESM.docx]

A Review on Imaging Techniques and Quantitative Measurements for Dynamic Imaging of Cerebral Aneurysm Pulsations

*November 3, 2020*

L. B. Stam^1*^, R. Aquarius^2^, G. de Jong^2^, C. H. Slump^3^, F. J. A. Meijer^4^, H. D. Boogaarts^2^

^1^ Technical Medicine, University of Twente, Enschede, The Netherlands. ^2^ Department of Neurosurgery, Radboud UMC, Nijmegen, The Netherlands. ^3^ Technical Medical Center, University of Twente, Enschede, The Netherlands. ^4^ Department of Radiology and nuclear medicine, Radboud UMC, Nijmegen, The Netherlands.

*Corresponding author. L. B. Stam

Department of Neurosurgery

Radboud University Medical Center

Geert Grooteplein-zuid 30

Internal post number 633

Nijmegen

The Netherlands

[Lotte.stam@radboudumc.nl](mailto:Lotte.stam@radboudumc.nl)

### Appendix C: Study population

***Table C.1****. Patient information. *Not mean but the median was given.*

|  | nr. of patients | age [mean, std, range] | gender (% male) | Heart rate [bpm] |
| --- | --- | --- | --- | --- |
| Kuroda | 18 | 63 ± 7,9 [49 76] | 17 | - |
| Firouzian | 14 | 59 ± 7 [47 70] | 36 | 77 ± 17 |
| Illies 2014 | 10 | 62 ± 8.2 [50 76] | -- | - |
| Illies 2016 | 12 | 59 ± 9 [43 71] | 67 | - |
| Kunitomi | 8 | 65 ± 7 [50 76] | 13 | 74 ± 11 [57 93] |
| Gu (irregular) | -- | -- | -- | - |
| Gu (regular) | -- | -- | -- | - |
| Dissaux | 11 | 57* [45 82] | -- | 87 |
| Oubel | 11 | 48 ± 17 [21 78] | 36 | - |
| Meyer | 10 | 61 ± 11 [45 81] | 20 | - |
| Karmonik | 7 | -- | -- | 79 ± 10 [66 97] |
| Kleinloog | 9 | 57 ± 11 [39 70] | 67 | 57 ± 11 [39 70] |

**Figure C.1**. Location per study. The upper bar visualizes the total. ICA: internal carotid artery; ACA: anterior cerebellar artery; MCA: middle cerebral artery; AcomA: anterior communicating artery; PcomA: posterior communicating artery; BA: Basilar artery; PICA: Posterior inferior cerebellar artery; OpthA: Opthalamic artery; PeriA: Pericallosal artery; AchoA: anterior
